# Hydroxyl Radical and Its Scavengers in Health and Disease

**DOI:** 10.1155/2011/809696

**Published:** 2011-07-17

**Authors:** Boguslaw Lipinski

**Affiliations:** Joslin Diabetes Center, Harvard Medical School, Boston, MA 02215, USA

## Abstract

It is generally believed that diseases caused by oxidative stress should be treated with antioxidants. However, clinical trials with such antioxidants as ascorbic acid and vitamin E, failed to produce the expected beneficial results. On the other hand, important biomolecules can be modified by the introduction of oxygen atoms by means of non-oxidative hydroxyl radicals. In addition, hydroxyl radicals can reduce disulfide bonds in proteins, specifically fibrinogen, resulting in their unfolding and scrambled refolding into abnormal spatial configurations. Consequences of this reaction are observed in many diseases such as atherosclerosis, cancer and neurological disorders, and can be prevented by the action of non-reducing substances. Moreover, many therapeutic substances, traditionally classified as antioxidants, accept electrons and thus are effective oxidants. It is described in this paper that hydroxyl radicals can be generated by ferric ions without any oxidizing agent. In view of the well-known damaging effect of poorly chelated iron in the human body, numerous natural products containing iron binding agents can be essential in the maintenance of human health. However, beneficial effects of the great number of phytochemicals that are endowed with hydroxyl radical scavenging and/or iron chelating activities should not be considered as a proof for oxidative stress.

## 1. Introduction

It is commonly believed that the *in vivo* damage of biomolecules is initiated by reactive oxygen species (ROS) in a process known as *oxidative stress*. However, oxidation reaction in biological systems may also occur via a nonradical pathway, for example, by hydrogen peroxide. In such cases, the products of its action are molecules that are enriched in one or more oxygen atoms that are generally considered to be markers of oxidative stress. Yet mere presence of extra oxygen does not tell us whether a given product is generated by a single- (free radical) or two-electron oxidation reaction. It should be emphasized that, according to the concept of oxidative stress, peroxidation and degradation of vulnerable biological molecules is caused by oxygen-centered radicals generated, in turn, by excessive blood oxygenation. However, this conclusion is based on studies of ischemia/reperfusion cases in which periods of prolonged hypoxia are followed by a sudden supply of oxygen. For example, in patients with extracorporeal circulation the appearance of lipid and/or nucleic acids oxidation products is accepted as signs of oxidative stress. However, a very important fact is being neglected, namely, that the most biologically active hydroxyl radicals are being predominantly formed during the period of ischemia in which oxygen is in short supply.

The uncritical acceptance of the fact that the presence of additional oxygen atom(s) in biomolecules and in their degradation products is a sign of oxidation led to a misleading definition of what is an antioxidant. According to principles of electrochemistry antioxidant must be endowed with a reductive property, but except ascorbic acid (vitamin C) and glutathione, there are very few natural substances that have reducing properties [1, 2]. Therefore, it is not surprising that ascorbic acid has not only failed to prevent consequences of oxidative stress [3] but on the contrary exhibited deleterious effects [4]. These facts present a paradox exemplified by a failure to demonstrate a significant effect of antioxidant therapies in diseases believed to be associated with oxidative stress, for example, atherosclerosis [5]. Several attempts were made to explain this problem in a series of publications [6–8]. Another line of evidence showing the lack of benefits from the use of antioxidants is their ineffectiveness in the prevention or treatment of hypertension [9]. By contrast, a diet rich in fruits and vegetables and whole grains protects cardiovascular system and lowers blood pressure by reducing free radical stress [10]. These important, albeit controversial, issues are being reviewed in this paper.

## 2. Generation and Properties of Hydroxyl Radicals

In living organisms there are two major reactive oxygen species, superoxide radical and hydroxyl radical that are being continuously formed in a process of reduction of oxygen to water. In the Haber-Weiss reaction hydroxyl radicals are generated in the presence of hydrogen peroxide and iron ions. The first step involves reduction of ferric into ferrous ion:

(1) $${\text{Fe}}^{3+}{+}^{∙}{\text{O}}_{2}^{-}\to {\text{Fe}}^{2+}+{\text{O}}_{2}.$$

The second step is the Fenton reaction:

(2) $${\text{Fe}}^{2+}+{\text{H}}_{2}{\text{O}}_{2}\;\to \;{\text{Fe}}^{3+}+{\text{OH}}^{-}\;{+}^{∙}\text{OH}.$$

The requirement of hydrogen peroxide in the Fenton reaction led to the misleading concept of oxidative stress that ignores the fact that hydroxyl radical (^∙^OH), known to be the most biologically active free radical, is formed *in vivo* under hypoxic conditions [11]. Moreover, this free radical can be generated *in vitro* under the reducing condition in the presence of ascorbic acid and iron ions. Even more intriguing is a discovery, presented in this paper, of the generation of hydroxyl radicals catalyzed by ferric ions without any additional redox agent, which can be considered as a special case of the Fenton reaction:

(3) $${\text{Fe}}^{3+}+{\text{HO}}^{-}\to {\text{Fe}}^{2+}\;{+}^{∙}\text{OH}$$

where one electron from the hydroxyl group of water is transferred to the ferric ion with the formation of a divalent iron and a *hydroxyl radical.*

 In addition to the essential role of hypoxic/reducing conditions for the *in vivo *generation of hydroxyl radicals, there is another important, although not generally recognized, factor namely free iron present in blood in the so called labile iron pool [12]. It was suggested some years ago that iron therapy might promote the formation of hydroxyl radicals thus contributing to atherosclerosis [13, 14]. In connection with this, it is of importance to note that excessive accumulation of stored iron is observed in atherosclerotic lesion [15] as well as in brains patients with neurological diseases [16]. In his paper with 136 references, Brewer reviewed the roles of both iron and copper in aging-related diseases [17]. More recently, another comprehensive review of the role of iron in degenerative diseases was published [18]. The close relationship between iron overload and pathogenesis of chronic disease can be explained in terms of the iron-induced hydroxyl radical generation and subsequent deposition of a fibrin-like material in various organs.

### 2.1. Hydroxyl Radical-Induced Polymerization of Human Fibrinogen

**Figure 1:**
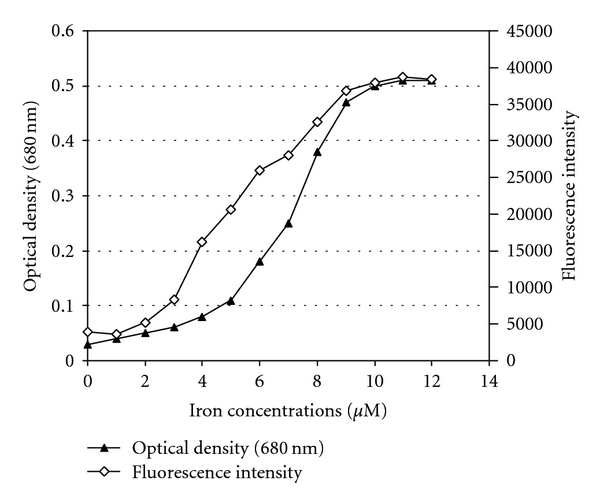
Effect of iron (ferric chloride) on fibrinogen aggregation, as measured by optical density, and on hydroxyl radical generation expressed as fluorescence intensity according to Manevich et al. (Radiat Res 1997; 148:580-91). A stock solution of 1 mM coumarin-3-carboxylic acid was diluted 1 : 2 with PBS, pH 7.4, and stored at room temperature before the use. Fifty-microliter aliquots of ferric chloride solution at various molar concentrations were added to black plastic microplate wells containing each 100 *μ*L of the coumarin reagent. After 2 min. incubation at RT fluorescence of the hydroxylation product was measured for one second in *PerkinElmer 1420 Multilabel Counter* at 350/450 nm of excitation/emission wavelength. The results presented in this figure show that the aggregation of fibrinogen is closely related to the extent of hydroxyl radicals generated in the system.

Fibrinogen is the most hydrophobic blood protein and thus it is very unstable in aqueous solutions being readily adsorbed on various surfaces [19]. In fact, the conversion of fibrinogen to fibrin by the action of thrombin renders fibrin monomers even more hydrophobic thus facilitating their spontaneous polymerization [20]. Moreover, exposure of purified fibrinogen to the ascorbic acid/cupric ion system results in the formation of an insoluble fibrin-like precipitate (neofiber) [21]. It was then serendipitously discovered that such a dramatic modification of fibrinogen is caused by hydroxyl radicals generated in the presence of ferric ions without any redox agent. As can be seen in Figure 1, the amount of precipitated fibrinogen is proportional to the number of hydroxyl radicals generated in the system.

Formation of insoluble aggregates under the influence of hydroxyl radicals is due to a limited reduction of *intra*molecular disulfide bridges followed by exposure of buried hydrophobic epitopes leading to the formation of extremely strong *inter*molecular bonds. Interestingly enough, acetylation of fibrinogen prior to the reaction with hydroxyl radicals rendered it susceptible to fibrinolysis [22]. It is very likely that this effect of salicylates is due to the introduction of a negative charge to the protein, thus diminishing hydrophobic interactions. An example of the effect of negative charge of sulfate groups is sulfitolysis of proteins that completely unfolds their polypeptide chains and yet maintains their complete solubility. It should be noted that fibrin-like deposits, which are remarkably resistant to proteolytic degradation, have been observed in numerous degenerative diseases such as atherosclerosis [23, 24], cancer [25], and diabetes [26] and in neurological disorders [27]. The same pathological conditions are characterized by excessive accumulation of poorly liganded iron, thus implicating a potential role of hydroxyl radicals in pathogenesis of these diseases [18].

## 3. Antioxidants and Free Radical Scavengers

There is a belief that polyunsaturated fatty acids (PUFAs) contribute to oxidative stress by providing a source of lipid peroxides. Yet by contrast to such expectations and according to epidemiological studies, PUFAs exert beneficial effects in those diseases that are known to be associated with free radical generation [28], and with complications of diabetes mellitus [29]. Another example of protective effect of unsaturated fatty acids is an intriguing phenomenon of the lack of signs of peroxidation in ringed arctic seals. These marine mammals hunt for fish by diving for periods of time up to 30 minutes, but, when their blood and organs are analyzed after they emerge from water and start breathing, no signs of oxidative stress are found [30]. Thus, it must be concluded that PUFAs present in their body fat protect these animals from the damaging consequences of prolonged hypoxia. This phenomenon can be explained in terms of a hydroxyl radical scavenging capacity of double bonds of polyunsaturated fatty acids.

Both omega-3 fatty acids and polyphenolic natural substances were shown to have protective effect in Alzheimer's disease [31] and in cancer [32] known to be linked to the persistent free radical stress [33], as well in diabetic nephropathy [34–36]. This apparent paradox can be explained by a strong affinity of hydroxyl radicals to double bonds that become reduced to single bonds [37]. In the case of unsaturated fatty acid (PUFA) the product formed is a hydroxy-fatty acid, which, in the presence of molecular oxygen, is further oxidized to aldehydes and ketones. This very important fact is an ultimate source of confusion, since these specific peroxidation products are considered to be markers of oxidative stress. 

In addition to the aforementioned substances, certain biologically active agents containing phenolic rings have the capacity to scavenge hydroxyl radicals by virtue of aromatic hydroxylation at the *ortho*position. One of such recently discovered scavengers is resveratrol shown to have a protective effect on ischemia-induced cerebral neuron damage [38]. Salicylates, that belong to the class of drugs with available *ortho*position at their phenolic ring, have also been demonstrated to protect against free radical injury *in vitro* [39] and *in vivo* [40]. The beneficial effect of salicylic acid in diabetes mellitus was first observed almost 100 years ago [41]. In addition, significant reduction of a risk of myocardial infarctions in diabetic patients has been attributed to the hydroxyl radicals scavenging activity of salicylates [42]. Cardiovascular consequences of excessive hydroxyl radical production in diabetic nephropathy may be explained in terms of their modification of fibrinogen molecule that becomes deposited in kidneys in the form of fibrin-like aggregates [43]. Protective effect against hydroxyl radical-induced damage in biological systems has been documented by the use of natural polyene and polyphenol class of substances, such as flavonoids, present mostly in fruits and vegetables [44, 45]. Also, hydroxyl radicals scavenging of double bonds present in quercetin may explain its beneficial effects on the modification of LDL molecule [46], similarly to the action of phenolic compounds in virgin olive [47]. Moreover, genistein, a naturally occurring isoflavone of soybeans, was recently documented to be an effective hydroxyl radical scavenger by virtue of the addition of hydroxyl groups to the double bonds of its aromatic rings [48].

It was recently shown in an elegant paper by Attia et al. [49] that proanthocyanidins, containing numerous phenolic rings, have a protective role in the abatement of doxorubicin-induced mutagenesis and cell proliferation changes in germinal cells of mice. Most recently, Acharya and his colleagues [50] have indicated that oxidative stress can have positive and negative effects on cellular proliferation, growth inhibition, and/or cell death. According to these authors determination of an *in vitro *antioxidant activity does not allow to predict potential benefits of plant-derived compounds, particularly in view of the reports showing that using antioxidants in clinical trials is associated with increased cancer incidences. Along the same line is Afanasev's argument that reactive oxygen species possess important function, namely, in molecular signaling in various pathophysiological processes [51].

The facts presented above clearly indicate that polyphenolic substances are endowed with both anti- and prooxidant properties. Antioxidant properties of polyphenols are based on their ability to be oxidized by, for example, hydrogen peroxide, to phenyl ketones (quinones) according to the following formula:

(4) Ph=CH–OH+H–O–O–H→Ph–HC=O+H–O–H.

At the same time these substances can scavenge hydroxyl radical (^∙^OH) by virtue of their addition to double bonds with the formation of a corresponding hydroxyl derivative:

(5) $$\text{R}–\text{CH=CH}–\text{R}+{\;}^{∙}\text{OH}\to \text{R}–\text{CH}–\text{(OH)}–{\text{CH}}_{2}–\text{R}.$$

Thus, a general conclusion can be drawn from these reactions that the difference between an oxidant and antioxidant is determined by the absence or presence of an electron in a carbon-carbon bond in an aromatic or aliphatic compound. It should be, however, emphasized that, in the case of polyphenols, only those with available *ortho*position in their phenolic rings will effectively scavenge hydroxyl radicals. Therapeutic effect of a mixture of proanthocyanides (*Oligol*), demonstrated in *in vitro* tests with breast cancer cells, can thus be explained by the presence of many such available sites in the structure of these polyphenols [52]. However, a particular redox property of a given polyphenol can only be properly assessed by methods using the 2′-deoxyguanosine assay with and without hydrogen peroxide [53]. 

## 4. Oxygen and Other Oxidants

Comparing oxidative stress to rusting of metals by oxygen led some researchers to a false conclusion that oxygen is deleterious to our health. It is being forgotten, however, that modern man lives in conditions under which we breathe less and less oxygen due to the industrial pollution and indoor living style. It was demonstrated by the largest epidemiological study conducted in Italy in the second half of this century that life expectancy was best correlated with high levels of respiratory indices, specifically vital capacity [54]. Moreover, oxygen therapy has been shown to have beneficial effect in various diseases. For example, tumor radiosensitivity is enhanced by increasing tumor oxygenation [55] that, in turn, may be explained by the effect of oxygen on the radiation-induced degradation of aggregated proteins [56]. The positive effect of hyperbaric oxygen in tumor radiation therapy was reviewed in two recent papers [57, 58].

In connection with this it should be noted that powerful oxidizing agent ozone was shown to selectively inhibit growth of human cancer cells [59] and to enhance the therapeutic effectiveness of 5-fluorouracil [60]. Contrary to adverse effects on human respiratory tract of ozone present in the polluted air, this strongly oxidizing agent was shown to be beneficial when properly administered into the circulation with ozone-treated blood [61]. Diabetic complications are also believed to be associated with oxidative stress; nevertheless diabetic ulcers were successfully healed with nothing else but a hyperbaric oxygen therapy [62]. Last but not least is the health beneficial influence of moderate exercise, particularly in the prevention of cardiovascular disease and consequences of aging. This effect is mediated by increased blood oxygenation, thus counterbalancing the damaging effect of hypoxia-induced generation of hydroxyl radicals. 

Another class of oxidants is the substances with disulfide groups in their structure that become reduced to thiols by hydroxyl radicals [63]. One such substance is lipoic acid (LPA) incorrectly classified as an antioxidant [64]. Reactivity of LPA is a classical example of oxidation without the addition of oxygen atom but just by a transfer of an electron from a substrate to the disulfide bond. The non-oxygen-assisted oxidation reaction is also exhibited by human serum albumin (HSA). This most abundant plasma protein contains sixteen *intra*molecular disulfide bonds that in the process of scavenging of hydroxyl radicals become reduced, unfolded, and aggregated. Relevant to this is a finding that aggregation of proteins is a result of their protective action in hypoxia/ischemic injury [65].

### 4.1. Selenium

Selenium is another oxidative component in diet, the role of which in health maintenance has generally been overlooked [66]. This metalloid is believed to be antioxidant due to its presence in the active centers of ROS-decomposing peroxidases and superoxide dismutases [67]. Selenium exists in soil mainly as a mineral sodium selenite in a wide spectrum of concentrations; thus its content in food products depends very much on a geographical area they are grown. Amongst organic forms of selenium the most abundant is selenocysteine that is now recognized as the 21st amino acid being present in about fourteen mammalian selenoproteins [68]. However, only sodium selenite and not the organic forms of selenium reacts rapidly with sulfhydryl groups (SH) of proteins (P) *oxidizing* them to disulfides according to the following reaction:

(6) $$\text{P}–{\left[\text{SH}\right]}_{2}+{\text{Na}}_{2}{\text{SeO}}_{3}\to \text{P}–\text{S}–\text{S}–\text{P}+\text{NaOH}+\text{SeO}$$

in which four-valent selenium is reduced to divalent oxide. Similarly, oxidative properties of selenite can be demonstrated by the reaction with ascorbic acid that becomes oxidized to dehydroascorbic acid. Thus, it is obvious that sodium selenite is an oxidant and not antioxidant as it is generally believed [69]. Oxidative properties of selenite have been investigated in various biological systems [70, 71]. This particular property may explain the augmentation of the function of natural killer cells, so important for cancer prevention and therapy [72], particularly in prostate cancer [73, 74].

Among the medical community there is an exaggerated concern about toxicity of selenium, with little or no attention being paid to its chemical form used in the treatment of cancer [73]. Yet data on selenium toxicity and anticancer activity was already available since 1994 [75]. In one study LD_50_ dose for selenite in rats was established to be 4.1 mg/kg body weight which is a thousand-fold greater than a maximum safe dose for humans [76]. In his review the author stated that such a dose is about 0.6 mg/day, and the toxic symptoms in the form of reversible hair loss and nails brittleness occur at the dose of 1 mg/day in a span of year or so. Importance of selenium in biological systems and its crucial role in health maintenance were extensively reviewed by Rayman [77] and more recently by two other authors [78, 79]. Data on selenium in relation to oxidative stress and health status can also be found in a paper by Brenneisen and colleagues [80]. Recent work has demonstrated that deficiencies in selenium result in increased viral pathogenicity and altered immune responses changing benign viruses into virulent ones [81]. Beneficial effects of sodium selenite in human colon cancer have also recently been reviewed [82].

## 5. Whole Foods or Food Supplements?

There are a number of recent publications concerning the importance of fruit and vegetable diet in the prevention of cardiovascular disease [83, 84]. It is well known that plant products contain vitamins such as A, B, C, and E, the beneficial effects of which have been attributed to their antioxidant properties. However, only very few of them are reducing agents and thus they cannot be classified as true antioxidants, and the remaining ones are either neutral or mild oxidants. Nevertheless, these natural phytochemicals, particularly polyphenols, have been shown to provide protection against many chronic diseases by virtue of their free radical scavenging properties [85–88]. Best known and already used in clinical practice is resveratrol, which neutralizes hydroxyl radicals by means of the aromatic hydroxylation reaction. Similar action against hydroxyl radicals is exerted by ferulic acid that belongs to the family of hydroxycinnamic acid with the chemical structure similar to curcumin [89]. Ferulic acid is found in leaves and seeds of many plants, especially in brown rice, whole wheat, oats, apples, artichokes, oranges, and pineapples. Curcumin (turmeric yellow) exerts anti-inflammatory activity, prevents atherosclerosis, and has protective action on brain [90]. This compound also binds cadmium and lead thus having additional benefits for human health. Likewise, extracts of *Sesamum indicum *seeds were shown to scavenge hydroxyl radicals and chelate metals [91].

Tapsell and her colleagues [92] have recently emphasized that the whole food, and particularly a combination of various natural food products, has a stronger health effect that any single biochemical or their combination. These authors conclude that the public may be better served by focusing on whole foods than on individual nutrients included in them. Moreover, there is no complete knowledge of food composition, and some effects may result from unidentified components. On the other hand, modification of certain food products, such as hydrogenation of vegetable oils, may be more harmful than the product to be substituted (e.g., butter) due to the generation of *trans* fatty acids. The concept of food* synergy* becomes particularly important in view of the lack of effect of many isolated compounds shown in clinical trials. As further emphasized by these authors, the acceptance of this concept has the potential to reduce the costs associated with an overdependence on pharmacological approaches to disease management. Another example of the beneficial effect of natural food products is isoprenoids, found in many fruits and plants including citrus (perillyl alcohol, geraniol), sage, spearmint, nutmeg (perillyl alcohol), basil (geraniol), lemon grass (farnesol and geraniol), and chamomile (farnesol) that are essential in the regulation of cell proliferation, apoptosis, differentiation, and lipid biosynthesis [93]. Flavonoids and food products containing them have potential positive effect on cardiovascular system, most likely due to their ability to chelate iron thus inhibiting the generation of reactive oxygen species [94].

Phenolic compounds and/or polyphenols are the important groups of compounds occurring in plants, comprising at least 8000 different known structures including simple phenols, phenolic acids, coumarins and isocoumarins, naphthoquinones, xanthones, stilbenes, flavonoids, and lignins. Flavonoids, constituting the most important polyphenolic class, are the natural substances exhibiting a wide range of biological effects including antibacterial, anti-inflammatory, antiallergic, antithrombotic, and vasodilatory actions [95]. There is also a plethora of other plants and herbs that contain polyphenolic substances that can potentially scavenge hydroxyl radicals and/or chelate free iron [96]. It is of interest to note that honey contains polyphenols endowed with antiproliferative potential [97]. In addition, *Euphorbia hirta*, an annual hairy plant native to India and Australia, has been reported to contain alkaloids, saponins, flavonoids, tannins phenolic acids, and amino acids. Traditionally, it is used in treatment of gastrointestinal disorders, bronchial and respiratory diseases, kidney stones, diabetes and in conjunctivitis. It also exhibits antipyretic, analgesic, antibacterial, anxiolytic, anthelmintic, antifertility, antispasmodic, antifungal, and anti-inflammatory activities [98]. *Crocus sativus *L. (saffron) is widely used in folk medicine, yet modern pharmacological studies have demonstrated that saffron extract or its active constituents have anticonvulsant, antidepressant, anti-inflammatory, and antitumor effects, as well as radical scavenging properties [99]. Phytochemicals, the structure of which indicates their scavenging ability of hydroxyl radicals, are listed in Table 1.

## 6. Conclusions

According to the traditional view of oxidative stress, its pathological consequences should be counteracted and/or prevented by antioxidants. However, clinical trials with such potent antioxidants as vitamins C and E gave conflicting if not negative results, thus questioning the concept of oxidative mechanism of the action of oxygen centered radicals. In fact, the most biologically reactive hydroxyl radical causes hydroxylation of various biomolecules by *reduction* of unsaturated bonds present in their structures. The same reductive principle operates in a number of natural substances endowed with aromatic and/or aliphatic double bonds making them effective scavengers of hydroxyl radicals. Numerous polyphenolic natural substances, commonly considered as antioxidants, are true *oxidants* due to the presence of electron acceptor groups in their molecules. Their beneficial effects support the importance of oxidative reactions in maintaining optimal health and/or preventing degenerative diseases. Oxidation of sulfhydryl groups on the cell surface of cancer cells by sodium selenite is another example of the importance of oxidative processes in health homeostasis.

## Figures and Tables

**Table 1:** Selected phytochemicals, their sources, and beneficial health effects.

Name	Source	Chemical class	Beneficial effect
Ajoene	Garlic	Polysulfide	Cancer, heart diseases
Apigenin	Fruits, vegetables	Flavonoid	Cancer
Aronia	Chokeberries	Anthocyanin	Cancer, inflammation
Caffeic acid	Coffee, cabbage	Flavonoid	Inflammation
Capsicum	*Solanaceae* plant	Capsaicin	Pain relief
Carvone	Caraway, dill	Terpenoid	Insecticide
Catechin	Chocolate, apples	Flavan-3-ol	Ischemic stroke
Chrysin	Passion flower	Flavones	Inflammation
Crocin	Honey	Polyphenols	Cold and flu
Devil's claw	African plant	Iridoid glycoside	Inflammation
Ellagic acid	Green tea	Cyclic polyphenol	Cancer
Farnesol	*Acacia* flowers	Aliphatic alcohol	Chemoprevention
Ferulic acid	Brown rice, apples	OH-cinnamic acid	Inflammation
Feverfew	*Asteracea* plant	Sesquiterpene lactone	Headaches
Galangin	Propolis	Trihydroxy-flavone	Antifungal
Ganoderic acid	Reishi	Triterpenoid	Breast cancer
Genistein	Lupin, soybeans	Isoflavone	Anticancer
GLA	Borage	Gamma-linolenic acid	Inflammation
Kaempferol	Tea, broccoli	Flavonoid	Cancer
Naringenin	Grapefruit	Flavonone	Hepatitis
Nasunin	Egg plant	Anthocyanin	Antiproliferative
Quercetin	Red onions	Polyphenol	Antihistamine,
Resveratrol	Red grapes	Trihydroxystilbene	Anti-aging
Sabinene	Caraway	Bicyclin monoterpene	Hypertension,
Safranal	Saffron	Aromatic ketone	Antidepressant
Salicin	Willow bark	Salicylic caid	Antipyretic
Silybin	Silymarin	Polyphenol	Liver diseases
Turmeric yellow	Indian plant	Curcumin	Diabetes, AD
